# Clinical Evaluation of a Pollen-Extract-Based Phytotherapy Compared to Conventional Therapies in Chronic Prostatitis and Chronic Pelvic Pain Syndrome

**DOI:** 10.3390/medsci13030186

**Published:** 2025-09-11

**Authors:** Marius Ivănuță, Dragoș Puia, Alin Adrian Cumpănaș, Ana-Maria Ivănuță, Veaceslav Groza, Cătălin Pricop

**Affiliations:** 1 “Grigore T Popa”, Faculty of Medicine, University of Medicine and Pharmacy, 700115 Iasi, Romania; marius.ivanuta@umfiasi.ro (M.I.);; 2 Department of Urology, “Dr. C.I. Parhon” Clinical Hospital, 700503 Iasi, Romania; grozaveaceslav@yahoo.com; 3 Center for Morphological and Spectroscopic Analysis of Urinary Stones” Michel Daudon”, 700503 Iasi, Romania; 4 Department XV, Discipline of Urology, “Victor Babes” University of Medicine and Pharmacy, 300041 Timisoara, Romania; 5 Emergency Department, “St. Spiridon” Emergency Clinical County Hospital, 700111 Iasi, Romania; ion.ana-maria@d.umfiasi.ro

**Keywords:** chronic pelvic pain syndrome, chronic prostatitis, pollen extract, phytotherapy, lower urinary tract symptoms, pain management, nonsteroidal anti-inflammatory drugs, alpha-blockers, quality of life

## Abstract

Background: Chronic prostatitis/chronic pelvic pain syndrome (CP/CPPS) is a prevalent condition characterized by pelvic pain and urinary symptoms with multifactorial aetiology. Standard treatments, including alpha-blockers, often have limited long-term effectiveness. This study aimed to evaluate the efficacy and safety of a standardized pollen extract (Deprox^®^ 500), alone or in combination with alpha-blockers, in reducing CP/CPPS symptoms and the need for rescue medication. Methods: This prospective, multicentre study included 207 male patients with CP/CPPS treated at two Romanian urology centres between January 2023 and January 2025. Patients were divided into three groups: Group A—alpha-blocker monotherapy; Group B—standardized pollen extract monotherapy; and Group C—combination therapy with standardized pollen extract and alpha-blocker. Symptom severity and treatment response were evaluated using the validated English versions of the NIH Chronic Prostatitis Symptom Index (NIH-CPSI), International Prostate Symptom Score (IPSS), and International Index of Erectile Function-5 (IIEF-5), all of which were translated into Romanian for use in this study. Results: Groups B and C both demonstrated significantly greater reductions in pelvic pain and urinary symptoms compared to Group A (*p* = 0.01), with marked improvements in NIH-CPSI and IPSS. Conclusions: A standardized pollen extract used alone or in combination with an alpha-blocker significantly improved CP/CPPS symptoms and reduced the need for NSAID rescue medication. These findings support its potential as a safe and effective therapeutic option.

## 1. Introduction

Chronic prostatitis is a prevalent condition with an estimated lifetime incidence of 1.8% to 8.2% [1,2]. It is essential to differentiate chronic prostatitis from other aetiologies of pelvic discomfort, including interstitial cystitis, benign prostatic hyperplasia (BPH), and alternative causes of dysuria. The National Institutes of Health (NIH) classifies prostatitis into four distinct categories: acute bacterial prostatitis (Category I), chronic bacterial prostatitis (Category II), chronic prostatitis/chronic pelvic pain syndrome (CP/CPPS) (Category III), and asymptomatic inflammatory prostatitis (Category IV). Category III is further divided into two subtypes: Type IIIA, characterised by the presence of inflammatory markers in the ejaculate; and Type IIIB, which lacks such inflammatory characteristics [3]. Since bacterial infections are confirmed in only 10% of cases diagnosed as prostatitis, most cases of chronic prostatitis are classified as Category III (inflammatory or non-inflammatory CP/CPPS) [4,5].

The pathogenesis of CP/CPPS remains poorly understood. Current evidence suggests a multifactorial origin involving a combination of infectious, neuromuscular, immune, endocrine, anatomical, and psychosocial factors in genetically or environmentally susceptible individuals [5]. Despite extensive research, optimal management of CP/CPPS remains elusive, with conventional therapies (alpha-blockers, NSAIDs, antibiotics, neuromodulators, physiotherapy) often providing only partial or transient relief. This therapeutic gap has driven interest in alternative, multifactorial approaches with favourable safety profiles. Standardized flower pollen extract, first noted in preliminary reports in 1995 [1], has since been supported by growing experimental and clinical evidence indicating sustained improvements in pain, urinary, and sexual function.

In the case of Category III CP/CPPS, which presents diagnostic challenges due to its heterogeneous symptomatology, the UPOINTS system, as outlined by Maeda et al., is frequently employed to tailor treatment based on symptomatology and underlying pathophysiological mechanisms [6]. Nevertheless, multimodal therapeutic strategies incorporating antibiotics, anti-inflammatory agents, alpha-blockers, neuromodulators, and physiotherapeutic interventions have demonstrated efficacy in symptom mitigation. Non-antibiotic therapies constitute a pivotal component of chronic prostatitis management, particularly in cases where antimicrobial regimens prove ineffective or unwarranted. Furthermore, phytotherapy and physiotherapy have garnered increasing recognition due to their favourable safety profiles, therapeutic efficacy, and high patient adherence. Emerging treatment modalities, including acupuncture, shockwave therapy, thermobalancing, and transcutaneous electrical nerve stimulation sono-electromagnetic therapy, have shown promise in alleviating the symptom burden associated with chronic prostatitis [7].

The present study aims to assess the efficacy and safety of a standardized pollen extract, administered either as monotherapy or in combination with an alpha-blocker, compared to alpha-blocker monotherapy—currently considered a standard of care. The analysis focuses on improvements in pain, urinary symptoms, and sexual function, using validated clinical tools including the NIH-CPSI (National Institutes of Health Chronic Prostatitis Symptom Index) [8], IPSS (International Prostate Symptom Score) [9], and IIEF-5 (International Index of Erectile Function-5) [10]. In addition, on-demand use of non-steroidal NSAIDs was recorded as a secondary outcome. By providing prospective, multicentre data from routine clinical practice, this study aims to clarify the potential role of phytotherapy in the personalized management of CP/CPPS.

## 2. Materials and Methods

### 2.1. Study Design and Setting

This was a prospective, non-randomized, open-label study conducted in two Romanian tertiary urology centres: the Clinic of Urology and Renal Transplantation at Dr. C.I. Parhon Hospital, Iași, and the Clinic of Urology at Timisoara County Hospital. The study was conducted between January 2023 and January 2025. All procedures complied with institutional ethical standards, and written informed consent was obtained from all participants prior to enrolment.

### 2.2. Participants and Eligibility Criteria

Patients diagnosed with chronic prostatitis/chronic pelvic pain syndrome (CP/CPPS), as defined by the 2016 EAU Guidelines and the NIH classification system [7], were considered for inclusion. Inclusion criteria required patients to report pelvic pain persisting for at least three of the preceding six months. Exclusion criteria included the presence of neurological disorders; pelvic malignancy; active urinary tract infections; and current or recent use of alpha-blockers, antimuscarinics, beta-3 agonists, or 5-alpha reductase inhibitors; and a known history or diagnosis of benign prostatic hyperplasia.

### 2.3. Treatment Groups and Interventions

Patients were assigned to treatment groups based on clinical presentation and therapeutic judgment. The three study groups were as follows:

- Group A: alpha-blocker monotherapy
- Group B: standardized pollen extract monotherapy
- Group C: combination therapy with standardized pollen extract and alpha-blocker

The standardized pollen extract administered in this study contained Graminex^®^ G63^®^ pollen extract (*Secale cereale*), vitamin B2, vitamin B1 and vitamin B12, commercially available as Deprox^®^ 500 (IDI Integratori Dietetici Italiani Srl, Aci Bonaccorsi, Italy) and known for its anti-inflammatory and antioxidative properties [11]. The treatment regimen followed the manufacturer’s recommendations: two tablets daily for 90 consecutive days. Alpha-blocker therapy consisted of tamsulosin 0.4 mg/day for the same duration. All patients were advised to use Diclofenac suppositories (100 mg) on an as-needed basis for acute pain episodes. Patients received detailed instructions on appropriate administration, including adherence to the maximum recommended daily dose, and were explicitly informed that NSAID use was reserved for situations of inadequate symptom control with the assigned therapy.

Treatment tolerability was assessed at each scheduled follow-up visit by directly questioning patients regarding adverse effects. Although no formal screening protocol for tamsulosin-related side effects was performed, none of the participants reported discontinuing the medication due to such events.

### 2.4. Outcome Measures

Symptom severity and treatment response were assessed using three validated, Romanian-translated questionnaires:

- NIH-CPSI: to evaluate pain, urinary symptoms, and quality of life
- IPSS: to assess lower urinary tract symptoms (LUTS)
- IIEF-5: to evaluate erectile function

NSAID consumption was systematically monitored throughout the study period. At the Month 1 and Month 3 visits, patients reported the number of diclofenac suppositories used since the previous assessment. Patients reported their use of diclofenac via structured questionnaires designed for this study.

These instruments were administered at baseline and after 12 weeks of treatment to allow for comparative evaluation of symptom progression.

### 2.5. Data Collection and Statistical Analysis

Demographic data, anthropometric measurements, and relevant comorbidities were extracted from electronic medical records. Data analysis was performed using IBM SPSS Statistics for Windows, Version 27.0 (IBM Corp., Armonk, NY, USA). Continuous variables were expressed as means ± standard deviation (SD), and categorical variables were presented as frequencies and percentages. Intergroup comparisons of categorical variables were conducted using the chi-square (χ^2^) test or Fisher’s exact test when expected counts were <5. The Mann–Whitney U test was used for continuous variables. A *p*-value < 0.05 was considered statistically significant.

The study was conducted in accordance with the principles of the Declaration of Helsinki and all applicable national regulations. The protocol received ethics committee approval prior to patient enrolment (approval no. 2572/20 March 2025). Written informed consent was obtained from all participants before inclusion in the study.

## 3. Results

**Figure 1:**
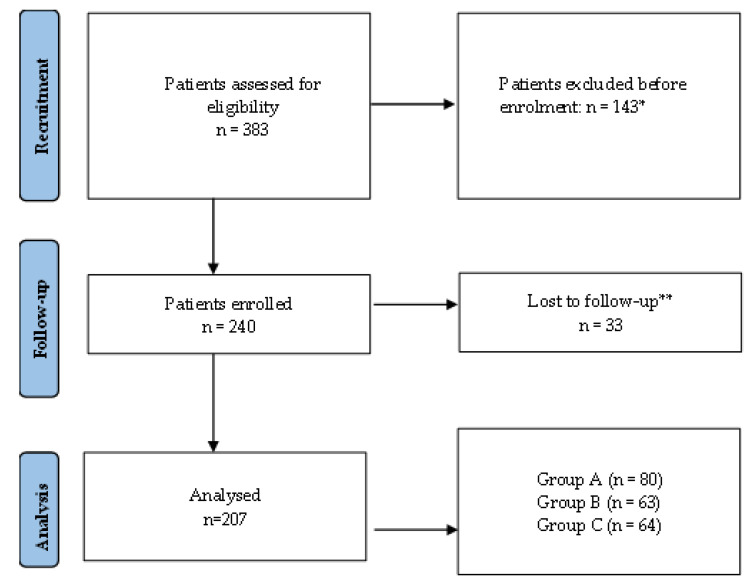
Flowchart of patient recruitment, exclusion, and follow-up in the study. * Reasons for exclusion: neurological disorders (*n* = 12); pelvic malignancy (*n* = 8); active urinary tract infection (*n* = 37); and prohibited medications (*n* = 86), including alpha-blockers (*n* = 35), antimuscarinics (*n* = 18), β3-agonists (*n* = 12), and 5-alpha reductase inhibitors (*n* = 21). ** Reasons for loss to follow-up: missed follow-up appointments or incomplete data.

As shown in Figure 1, the flowchart summarizes patient recruitment, exclusion, and follow-up in the study. A total of 383 patients were assessed for eligibility. Of these, 143 were excluded according to predefined exclusion criteria. The remaining 240 patients were enrolled, 33 of whom were lost to follow-up. The final analysis included 207 patients (Group A—80, Group B—63, and Group C—64). Of these, 154 patients (74.4%) were treated at the Clinic of Urology and Renal Transplantation, Dr. C.I. Parhon Hospital in Iași, and 53 patients (25.6%) were treated at the Clinic of Urology, Timișoara County Hospital.

The mean age of participants was 51.44 years (±9.13), with no statistically significant differences between groups (*p* = 0.62). Specifically, the mean age was 52.23 years (±8.08) in Group A, 50.84 years (±10.31) in Group B, and 52.23 years (±8.03) in Group C.

The mean body mass index (BMI) across the whole sample was 27.86 kg/m^2^ (±5.24), with no significant differences between groups (*p* = 0.33). The mean BMI of each group was as follows: Group A—28.48 kg/m^2^ (±5.91), Group B—27.18 kg/m^2^ (±4.76), and Group C—27.76 kg/m^2^ (±4.75).

Table 1 summarizes the patients’ demographic characteristics, comorbidities, and selected lifestyle factors.

Table 2 presents baseline characteristics and post-treatment outcomes across the three study groups. At baseline, no statistically significant differences were observed in IPSS, NIH-CPSI, IIEF-5, pelvic pain, urination score, or QoL (*p* > 0.05), indicating comparable symptom burden and functional status at study entry.

Following treatment, all groups demonstrated clinically relevant improvement across most endpoints. The reduction in total IPSS was statistically significant between groups (*p* = 0.006), with post-hoc comparisons indicating that Group B and Group C achieved lower scores than Group A, reflecting superior improvement of lower urinary tract symptoms. Across the whole sample, mean IPSS decreased from 18.55 ± 8.26 to 12.48 ± 5.49.

NIH-CPSI total scores also improved significantly across groups (*p* = 0.003). The highest reduction was observed in Group B, followed by Group C, with the difference driven predominantly by the pain subdomain (*p* < 0.001).

Erectile function, as assessed by IIEF-5, showed modest intragroup improvements; however, no statistically significant differences were detected between groups post-treatment (*p* = 0.98). Similarly, changes in the urination subscore were not statistically significant between groups (*p* = 0.27), although numerical improvements were seen in all arms.

QoL scores decreased numerically in all groups, consistent with an improvement in patient-perceived well-being, given that lower scores indicate better QoL on this scale. Intergroup differences were significant (*p* = 0.004), with Group B and Group C reporting the lowest post-treatment scores, suggesting greater overall treatment benefit in these arms.

The statistical analysis of post-treatment outcomes revealed several significant differences between study groups. These differences, assessed using the Tukey HSD test, are summarized in Table 3, which presents the pairwise comparisons for IPSS, NIH-CPSI, IIEF-5, pain score, urination score, and quality of life.

As shown in Table 4, NSAID requirements decreased from baseline to Month 3 in all study groups. The reduction was higher in the group receiving extract of Graminex 500 alone and in the combination therapy group, while the decrease was smaller in the tamsulosin monotherapy group. Both extract of Graminex 500-containing regimens demonstrated significantly larger reductions compared with tamsulosin alone (*p* = 0.03 and *p* = 0.02, respectively), whereas no statistically significant difference was observed between the two extract-containing groups (*p* > 0.90).

## 4. Discussion

Managing CP/CPPS continues to pose a significant clinical challenge, largely due to the absence of a clearly defined aetiology and the modest effectiveness of conventional treatments. In clinical practice, alpha-blockers and NSAIDs remain standard options but are often associated with incomplete symptom control and frequent relapse. Antibiotic use, although widespread, is empirically applied in most cases and rarely supported by positive cultures or markers of infection. As a result, therapeutic strategies that address non-infectious mechanisms such as inflammation and pelvic neuromuscular dysfunction have gained increasing attention [12,13].

Phytotherapeutic agents, and particularly pollen-based formulations, have emerged as promising alternatives in this context. Several clinical studies have reported that standardized pollen extracts can reduce symptom burden and improve patient-reported outcomes in CP/CPPS. For instance, Cai et al. showed that a four-week course of pollen extract combined with vitamins led to greater reductions in NIH-CPSI scores than ibuprofen [9]. In another randomized study, Macchione et al. found that a similar extract produced significantly better results than *Serenoa repens*, both in pain relief and urinary symptom scores. These findings suggest a potential therapeutic role for pollen extracts in targeting local inflammatory pathways [13].

Moreover, preliminary biomarker data support this hypothesis. Cai et al. demonstrated a significant post-treatment decrease in IL-8 levels among patients treated with pollen extract, reinforcing its proposed anti-inflammatory mechanism of action. Such evidence underscores the rationale for integrating phytotherapeutic agents into the management of CP/CPPS, particularly when conventional options fail or are poorly tolerated [14].

Among the three treatment strategies explored in our study, the most consistent symptom improvements were observed in patients who received the standardized pollen extract—either alone or in combination with an alpha-blocker. Those who received pollen extract as monotherapy reached the lowest mean NIH-CPSI scores at follow-up and reported the most substantial reduction in pain intensity [15].

These findings align closely with previous work by Cai et al., who showed that a four-week course of pollen extract with vitamins reduced the NIH-CPSI from 25.2 to 16.1, with pain scores decreasing from 11.8 to 6.6 [14]. In our cohort, although baseline values were slightly higher, the post-treatment results fell within a similar range, reinforcing the reproducibility of the therapeutic effect. Quality of life improvements followed a similar trajectory, again favouring the phytotherapeutic intervention.

Interestingly, patients who received the combination of pollen extract and alpha-blockers did not show clearly superior results compared to the pollen monotherapy group. Their symptom scores improved, particularly in pain and QoL domains, but not beyond the level achieved by phytotherapy alone. This pattern is not unique to our data. A similar conclusion was drawn in the trial by Aoki et al., where the combination of tamsulosin and pollen extract produced slightly better outcomes than tamsulosin alone, but not significantly beyond those achieved with pollen extract as a single agent [16].

Taken together, these findings raise an important clinical consideration: The additive value of alpha-blockers may be limited, at least in the short term, when a pollen-based formulation is already in use. While combination therapy was effective and safe, the data suggest that the primary driver of symptom improvement may be the phytotherapeutic component.

Urinary symptoms, assessed using the IPSS, improved across all groups. Patients who received pollen extract, either alone or in combination, experienced higher reductions compared to those treated with alpha-blockers only. The average IPSS score dropped from 18.5 at baseline to around 11 post-treatment in the phytotherapy groups, while the alpha-blocker group remained closer to 14. This again mirrors the results seen in Macchione et al., where pollen extract produced a more substantial reduction in IPSS than *Serenoa repens* after six weeks of treatment [13].

An important consideration in phytotherapy research is the placebo effect, which has been well documented in CP/CPPS trials. However, Cai et al. emphasized that, despite the presence of placebo responses, the group treated with pollen extract consistently showed clinically significant improvements, supporting the inclusion of pollen-based therapies in the EAU guidelines for managing chronic prostatitis [7,14].

Clinical evidence accumulated over the past decades supports the efficacy of pollen extract in alleviating symptoms associated with CP/CPPS. As early as 1993, Rugendorff et al. reported substantial improvements in both QoL and LUTS in up to 78% of patients treated with pollen extract, alongside a notably high tolerability rate of 97% [17]. These early findings have been reinforced by more recent data. A comprehensive review by MacDonald et al., which included 444 male patients, confirmed that pollen extract is not only well tolerated but also consistently effective in improving urological symptoms across a variety of clinical settings [18]. Similarly, Iwamura et al. demonstrated symptom relief in 78% of patients, with significant reductions in NIH-CPSI scores and notable improvements in pain perception and overall QoL [19]. Supporting these results, Wagenlehner et al. found that patients receiving pollen extract for three months experienced greater improvements in pain, symptoms, and quality of life compared to placebo [20]. Further validation came from Elist’s randomized, double-blind, placebo-controlled trial, which confirmed the superiority of pollen extract over placebo in terms of both symptom reduction and pain control [21].

In our study, the evaluation of erectile function using the IIEF-5 questionnaire revealed varying outcomes across the treatment groups. Deprox^®^ 500 monotherapy (Group B) did not result in significant improvements compared to alpha-blocker monotherapy (Group A) (*p* = 0.98). While previous research demonstrated that pollen extract exerts anti-proliferative effects on prostatic cells and may positively influence sexual health outcomes, our findings differ, indicating a limited impact of pollen extract alone on erectile function [15,22,23].

QoL, a key outcome in CP/CPPS management, improved across all treatment arms, with the most pronounced improvement observed in the group receiving combination therapy. In this group, the NIH-CPSI QoL subscore decreased from 7.33 to 5.55. The pollen monotherapy group showed a comparable trend, with scores dropping from 6.84 to 5.59, while the alpha-blocker group exhibited only a minimal change (7.55 to 7.36).

Importantly, the between-group comparison revealed a statistically significant difference (*p* = 0.004), indicating that interventions including pollen extract were associated with better perceived quality of life. These findings are consistent with those reported by di Pasquale et al., who noted similar QoL improvements in patients treated with pollen-based formulations, particularly in cases involving chronic pelvic discomfort following urological surgery [23]. Given the complex psychosomatic dimensions of CP/CPPS, such improvements—though modest in absolute terms—may translate into meaningful benefits in day-to-day functioning and emotional well-being.

Beyond subjective symptom scores, the reduction in NSAID consumption across treatment arms provides additional insight into clinical efficacy. By Month 3, patients treated with pollen extract—either alone or in combination—showed a greater decrease in NSAID use compared to those in the alpha-blocker group. Specifically, the reduction was 164.84 mg (±141.05) in the combination group and 161.84 mg (±192.54) in the monotherapy group, versus only 89.37 mg (±177.30) in the alpha-blocker group. These differences were statistically significant (Group A vs. B, *p* = 0.03; Group A vs. C, *p* = 0.02), while no difference was observed between the two pollen-based interventions. This suggests that pollen extract may not only alleviate symptoms directly but also reduce patients’ reliance on adjunctive pharmacologic pain management—an important clinical consideration given the risks associated with long-term NSAID use.

These findings align with existing literature on the anti-inflammatory and antioxidative properties of pollen extracts. Loschen et al. demonstrated that rye pollen extract inhibits prostaglandin and leukotriene synthesis, mimicking the anti-inflammatory effects of NSAIDs like diclofenac [24]. This likely accounts for the reductions in pain and urinary symptoms observed in the present study. Furthermore, other authors reported that pollen extract modulates cytokine levels, leading to reductions in IL-1β, IL-6, and TNF-α—key mediators of prostatic inflammation [25,26].

The inhibition of 5α-reductase by pollen extract, as previously described by Muraca, offers a plausible mechanistic explanation for the observed improvements in urinary symptoms [27]. By reducing the conversion of testosterone to dihydrotestosterone, pollen extract limits prostatic enlargement and alleviates bladder outlet obstruction, leading to enhanced urinary flow and symptom relief. The significant decline in IPSS scores in Group C further supports this mechanism.

In the broader context of CP/CPPS management, our findings contribute to the growing body of evidence supporting the potential role of phytotherapeutic agents in alleviating symptoms associated with this complex condition. Although the placebo effect remains an important factor to consider in trials involving plant-based therapies [28,29], the observed improvements in symptom scores and NSAID use reduction across groups receiving pollen extract suggest a relevant clinical benefit that merits further exploration. Additionally, the treatment was well tolerated, with no reported adverse effects, reinforcing its safety profile within the limits of the study duration.

Several limitations should be acknowledged. The lack of long-term follow-up limits our ability to assess the persistence of therapeutic effects beyond the three-month period. Furthermore, reliance on self-reported outcomes may introduce bias, particularly in a condition characterized by fluctuating symptoms and strong subjective components. Importantly, the absence of a placebo-controlled arm and the non-randomized design restrict the extent to which treatment effects can be confidently attributed to the interventions themselves. Another limitation of this study is that the Romanian versions of NIH-CPSI, IPSS, and IIEF-5 questionnaires have not undergone a prior formal validation.

## 5. Conclusions

In this prospective comparative study, standardized pollen extract—administered as monotherapy or in combination with alpha-blockers—was associated with measurable improvements in symptom severity, pain levels, and quality of life among patients with chronic prostatitis/chronic pelvic pain syndrome. Additionally, a decrease in NSAID consumption was observed in both pollen-treated groups, suggesting a potential role in reducing the burden of adjunctive pharmacologic therapy.

## Figures and Tables

**Table 1:** Baseline demographic characteristics, comorbidities, and lifestyle factors of the study population.

Parameter		Whole Sample (*n* = 207)		Group A (*n* = 80)		Group B (*n* = 63)		Group C (*n* = 64)		*p*
		*n*	%	*n*	%	*n*	%	*n*	%	
Hypertension	Yes	122	58.9	49	63.1	34	54	39	60.9	0.63
	No	85	41.1	31	38.8	29	46	25	39.1	
Diabetes	Yes	62	30	28	35	17	27	17	26.6	0.45
	No	145	70	52	65	46	73	47	73.4	
Dyslipidaemia	Yes	89	43	32	40	29	46	28	43.8	0.76
	No	118	57	48	60	34	54	36	56.2	
History of endourological surgery	Yes	47	22.7	15	18.8	16	25.4	16	25	0.55
	No	160	77.3	65	81.2	47	74.6	48	75	
Smoking	Yes	70	33.8	23	28.7	23	36.5	24	37.5	0.47
	No	137	66.2	57	71.2	40	63.5	40	62.5	
Alcohol consumption	Yes	65	31.4	26	32.5	22	34.9	17	26.6	0.57
	No	142	68.6	54	67.5	41	65.1	47	73.8	
More than one sexual partner last year	Yes	47	22.7	20	25	14	22.2	13	20.3	0.79
	No	160	77.3	60	75	49	77.8	51	79.7	

**Table 2:** Baseline Scores and Post-Treatment Changes.

Characteristics	Whole Sample	Group A	Group B	Group C	*p*
IPSS–Baseline ±SD	18.55 ± 8.26	17.68 ± 7.23	19.44 ± 9.75	18.75 ± 7.89	0.43
IPSS–Posttreatment ±SD	12.48 ± 5.49	13.96 ± 5.7	11.86 ± 5.02	11.23 ± 5.30	0.006
NIH-CPSI– Baseline ±SD	24.56 ± 7.01	25.55 (±6.88)	23.26 ± 6.44	24.60 ± 7.57	0.15
NIH-CPSI –Posttreatment ±SD	17.03 ± 6.36	19.72 ± 6.80	16.31 ± 5.02	17.03 ± 6.47	0.003
IIEF-5–Baseline ±SD	16.16 ± 5.33	16.43 (±4.83)	15.60 ± 5.74	16.39 ± 5.45	0.60
IIEF-5 –Posttreatment ±SD	17.37 ± 5.27	17.30 ± 4.64	17.44 ± 5.86	17.39 ± 5.46	0.98
Pain Score–Baseline ±SD	14.23 ± 3.42	14.64 ± 3.52	13.71 ± 3.13	14.22 ± 3.5	0.27
Pain Score –Posttreatment ±SD	7.08 ± 3.27	8.59 ± 3.36	6.05 ± 2.14	6.20 ± 3.36	<0.001
Urination Score–Baseline ±SD	6.67 ± 3.37	6.43 ± 3.19	6.41 ± 3.35	7.22 ± 3.58	0.29
Posttreatment ±SD	6.29 ± 3.98	6.54 ± 4.58	5.62 ± 3.31	6.66 ± 3.76	0.27
QoL– Baseline ±SD	7.27 ± 2.82	7.55 ± 2.76	6.84 ± 2.38	7.33 ± 3.26	0.32
QoL–Posttreatment ±SD	6.26 ± 3.81	7.36 ± 4.04	5.59 ± 3.48	5.55 ± 3.55	0.004

SD—Standard deviation, IPSS—International Prostate Symptom Score, NIH-CPSI—National Institutes of Health Chronic Prostatitis Symptom Index, IIEF-5—International Index of Erectile Function—5-item version, QoL—Quality of life.

**Table 3:** Pairwise comparisons between study groups for post-treatment scores (Tukey HSD test).

Score	A vs. B	B vs. C	A vs. C
IPSS	0.05	0.79	0.008
NIH-CPSI	0.004	0.79	0.028
IIEF-5	0.98	0.84	0.99
Pain score	<0.001	0.95	<0.001
Urination Score	0.35	0.30	0.98
QoL score	0.01	0.95	0.01

**Table 4:** Changes in NSAID requirements from baseline to Month 3 across treatment groups.

Group	NSAID Use M1 Mean ± SD	NSAID Use M3 Mean ± SD	M3–M1 Mean ± SD	95% CI	Comparisons
Group A	380 ± 164.84	290.62 ± 162.86	89.37 ± 177.30	49.91–128.83	vs. B — *p* = 0.03 vs. C — *p* = 0.02
Group B	352 ± 216.91	190.47 ± 137.62	161.84 ± 192.54	113.41–210.39	vs. C — *p* > 0.90 vs. A — *p* > 0.03
Group C	292.18 ± 183.70	127.34 ± 110.16	164.84 ± 141.05	126.61–200.07	vs. A — *p* = 0.02 vs. B — *p* > 0.90

NSAID use–average monthly NSAID consumption (mg) at Month 1 (M1) and Month 3 (M3). M3–M1 represents the difference between the two time points. Bonferroni-adjusted *p*-values are reported.

## Data Availability

The original contributions presented in this study are included in the article. Further inquiries can be directed to the corresponding author.

## Abbreviations

The following abbreviations are used in this manuscript:

BPH	Benign Prostatic Hyperplasia
NIH	National Institutes of Health
CP/CPPS	Chronic Nonbacterial Prostatitis/Chronic Pelvic Pain Syndrome
NSAIDs	Nonsteroidal Anti-Inflammatory Drugs
EAU	European Association of Urology
IIEF-5	The International Index of Erectile Function-5
IPSS	International Prostate Symptom Score
QoL	Quality of Life
